# Screening for type 2 diabetes and periodontitis patients (CODAPT-My©): a multidisciplinary care approach

**DOI:** 10.1186/s12913-022-08429-w

**Published:** 2022-08-13

**Authors:** Aznida Firzah Abdul Aziz, Tuti Ningseh Mohd-Dom, Norlaila Mustafa, Abdul Hadi Said, Rasidah Ayob, Salbiah Mohamed Isa, Ernieda Hatah, Sharifa Ezat Wan Puteh, Mohd Farez Fitri Mohd Alwi

**Affiliations:** 1 Department of Family Medicine, Faculty of Medicine, National University of Malaysia, Jalan Yaacob Latif, Kuala Lumpur, 56000 Malaysia; 2 Department of Family Oral Health, Faculty of Dentistry, National University of Malaysia, Jalan Raja Muda Abdul Aziz, Kuala Lumpur, 50300 Malaysia; 3 Department of Internal Medicine, Faculty of Medicine, National University of Malaysia, Jalan Yaacob Latif, Kuala Lumpur, 56000 Malaysia; 4 Department of Family Medicine, Kulliyyah of Medicine, International Islamic University of Malaysia, Kuantan, Pahang 25150 Malaysia; 5 Oral Health Programme, Ministry of Health, Malaysia, Level 5, Presint 1, Putrajaya, 62590 Malaysia; 6 Klinik Kesihatan Bandar Botanic, Ministry of Health, Malaysia, Klang, Selangor 42000 Malaysia; 7 Faculty of Pharmacy, National University of Malaysia, Jalan Raja Muda Abdul Aziz, Kuala Lumpur, 50300 Malaysia; 8 Department of Community Health, Faculty of Medicine, National University of Malaysia, Jalan Yaacob Latif, Kuala Lumpur, 56000 Malaysia; 9 Hospital Ampang, Ministry of Health of Health, Malaysia, Jalan Mewah Utara, Ampang, Selangor 68000 Malaysia

**Keywords:** Care pathway, Primary healthcare, Dental, Periodontitis, Diabetes, Prediabetes

## Abstract

**Background:**

The practice of referring diabetic patients for dental intervention has been poor despite awareness and knowledge of the oral health effects of diabetes. Likewise, dentists treating patients receiving diabetes treatment are rarely updated on the glycaemic status and as a result, the opportunity for shared management of these patients is missed. This study aimed to provide a standardised care pathway which will initiate screening for diabetes from dental clinics and link patients with primary care for them to receive optimised care for glycaemic control.

**Method:**

A Modified Delphi technique was employed to obtain consensus on recommendations, based on current evidence and best care practices to screen for diabetes among patients attending dental clinics for periodontitis. Expert panel members were recruited using snowball technique where the experts comprised Family Medicine Specialists (5), Periodontists (6), Endocrinologists (3) and Clinical Pharmacists (4) who are involved in management of patients with diabetes at public and private healthcare facilities. Care algorithms were designed based on existing public healthcare services.

**Results:**

The CODAPT^©^ panel recommends referral to primary care for further evaluation of glycaemic status if patients diagnosed with periodontitis record fasting capillary blood glucose levels ≥ 5.6 mmol/L. Intervention treatment options for prediabetes are listed, and emphasis on feedback to the dental healthcare team is outlined specifically.

**Conclusion:**

The CODAPT^©^ care pathway has the potential to link dental clinics with primary care for diagnosis and/or optimised treatment of prediabetes/diabetes among patients receiving periodontitis treatment.

## Background

Diabetes mellitus is a disease linked to a spectrum of other non-communicable diseases (NCDs) and complications and providing a holistic management for patients with this condition involves a multidisciplinary involvement. The rising prevalence and incidence of diabetes in Malaysia calls for efforts to increase awareness among stakeholders to focus on early detection and to optimise resources in managing patients at risk of diabetes. The National Health and Morbidity Survey in 2019 reported that 8.9% of the 18.3% diabetics were unaware that they had diabetes (i.e., FBS > 7.0 mmol/L) [1]. The bidirectional link between diabetes and periodontitis therefore presents an ideal logistic opportunity for detection of both diseases among patients attending healthcare services at earliest possible opportunity [2].

Periodontitis is also a major public health problem. It has significant impacts on individuals by reducing their quality of life and dental care can consume between 10–15% of the total allocation towards healthcare [3, 4]. Current estimates projected that periodontitis affects 20–50% of the world population while the average prevalence of severe periodontitis globally is at 9.2% [5]. Locally, periodontal disease prevalence declined between 1990 (92.8%) and 2000 (87.2%); however, a sharp rise was observed in the 2010 National Oral Health Survey for Adults (NOHSA) (94.0%) [6, 7]. The extremely high prevalence of periodontitis among adults is also challenged by the poor oral healthcare utilisation practices among Malaysian adults where only 13.2% reportedly saw a dentist within the last 12 months as highlighted in the National Health and Morbidity Survey in 2019 [8].

As an added concern, the prevalence, severity, and progression of periodontal diseases are significantly increased in patients with diabetes due to the poor glycaemic control [9] and insulin resistance [10]. Moreover, patients with diabetes are 2 to 3 times more likely to have periodontitis compared to non-diabetic patients, and this is related to long term metabolic control and duration of diabetes [11]. Patients with diabetes have greater risk of developing more severe medical complications including retinopathy, nephropathy, cardiovascular complications and even risk of cardiorenal mortality [10]. There are evidence demonstrating the effect of periodontal therapy on the HbA_1c_ level. Non-surgical periodontal treatment results in a modest reduction of -0.36% of HbA_1c_ [12] and a statistically significant reduction in HbA_1c_ levels at 3 months, with a lower reduction at 6 months, ranging from − 0.27% (95% CI: − 0.46, − 0.07, p = 0.007) to − 1.03% [13]. A systematic review on randomised clinical trials found that periodontal therapy significantly contributed to glycaemic control in T2DM patients and there was a greater reduction in HbA_1c_ after periodontal therapy for patients with higher baseline HbA_1c_ level [14]. Periodontal therapy, therefore, has the potential to reduce mortality in T2DM patients and may be a useful adjunct to medical management of diabetes [15].

The oral healthcare system in Malaysia operates mostly as a separate entity from the primary healthcare system where there is no established pathway for bilateral case referrals. The oral health status of diabetes patients attending primary healthcare facilities is not monitored, and the lack of awareness regarding the bidirectional relationship between periodontal diseases and diabetes mellitus among both healthcare personnel and patients in Malaysia compounds the problem [16]. In the Malaysian public health system, primary healthcare and dental care services are provided by 1114 health centres, of which a total of 577 dental clinics are based within the same premises as these health centres [17]. The geographical location of the public primary healthcare and dental care services, particularly periodontal specialist care, which is within the community health centre complex presents an opportunity for providing coordinated care for diabetic patients with periodontitis or for addressing oral healthcare surveillance of patients with diabetes [18]. Yet, there remains a lack of coordination between medical and dental professionals; this situation is not unique to Malaysia as healthcare systems in the UK and most parts of the world are reported to have faced similar experiences [19].

Hence, this study aimed to develop a care pathway to provide a guide for a multidisciplinary management plan in the community for periodontitis patients who are detected to have diabetes mellitus as well as those at risk for diabetes, and vice versa. The CODAPT^©^ aims to facilitate the link between dental and primary healthcare teams in managing the patients through a comprehensive and coordinated manner, without putting additional burden or inconvenience on the patients.

## Methodology

### Expert panel selection

**Fig. 1:**
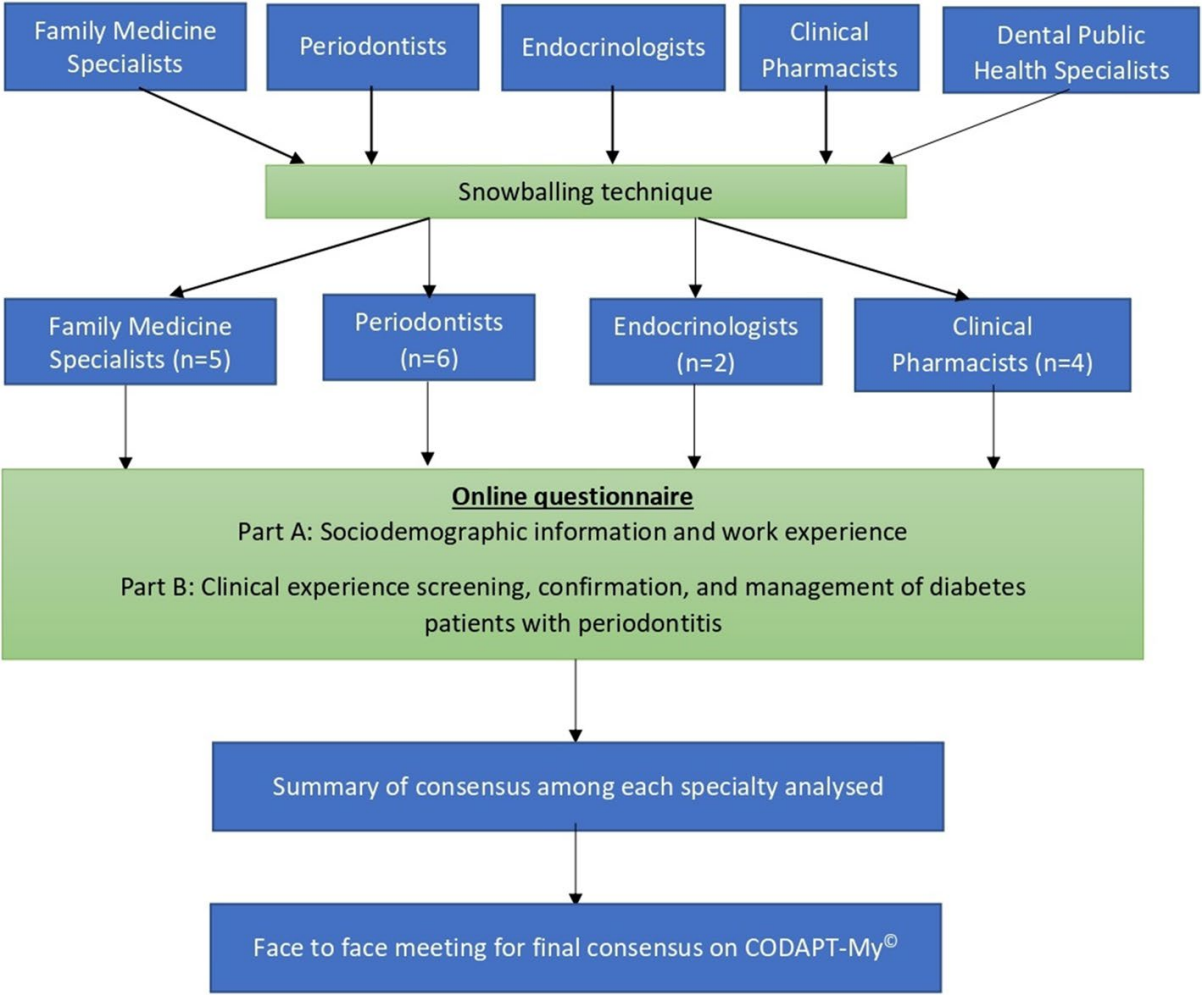
Study flow chart

The research team discussed and identified all the disciplines involved in healthcare provision for periodontitis patients with diabetes in an ideal seamless healthcare set-up. The team agreed that the expert panel members should include Periodontists, Family Medicine Specialists, Endocrinologists and Clinical Pharmacists. All members of the expert panel are clinicians from the public and private sectors including those at university health care facilities, and were actively involved in providing care for patients with diabetes. Members of the expert panel were recruited using snowball technique (Fig. 1) through which multidisciplinary experts who were directly involved in the management of patients with diabetes or periodontitis or both were identified.

### Panel recruitment

Panel members were invited to participate in the discussion via personalised email invitation; additionally, reminders to respond in relation to their willingness to participate were also sent out via email. If no response was received after two email reminders, they were considered as non-responders.

### Conduct of data collection

All respondents who agreed to participate received an online link to the questionnaire (Google form). The questionnaire contained a brief introduction and summary of the diabetes-periodontitis link and related references from published credible sources. The latter was provided as full-length publications which the respondents were able to download and read at their convenience as they answered the questionnaire. The first section of the questionnaire gathered the sociodemographic background and work experience of the respondents. The second part of the questionnaire was on the respondents’ clinical experience in the screening, confirmation, and management of diabetes patients with periodontitis. The questionnaire addressed issues related to confirmation protocol of diabetes mellitus based on the clinical practice guidelines issued by the Ministry of Health Malaysia. A Modified Delphi technique was employed to achieve consensus on responses which were not unanimous. A face-to-face meeting was conducted on 17th October 2019 to finalise the care pathway and to endorse the final document. The finalised document was then shared with the expert panel members for checking and endorsement.

### Data analysis

Data entry and analysis to calculate the descriptive statistics was performed using Microsoft Excel.

## Results

### Background of expert panel members

A total of 17 experts agreed to participate. The background of the experts is presented in Table 1. The experts had a minimum of 10.3 (SD4.9) years of experience in their clinical field and majority were from the public sector.

**Table 1:** Background of expert panel members

Clinical expertise	N	Gender	Sector	Age (Mean SD)	Years of service (Mean, SD)
Family Medicine Specialists and Endocrinologists	7	Female 5 Male 2	Public 5 Private 2	46.6, 6.8	13.6, 10.5
Periodontists	6	Female 4 Male 2	Public 5 Private 1	48.8, 7.6	11.2, 7.5
Clinical Pharmacists	4	Female 2 Male 2	Public 4	35.8, 4.9	10.3, 4.9

### The CODAPT-My© algorithm for the dental practitioner

**Fig. 2:**
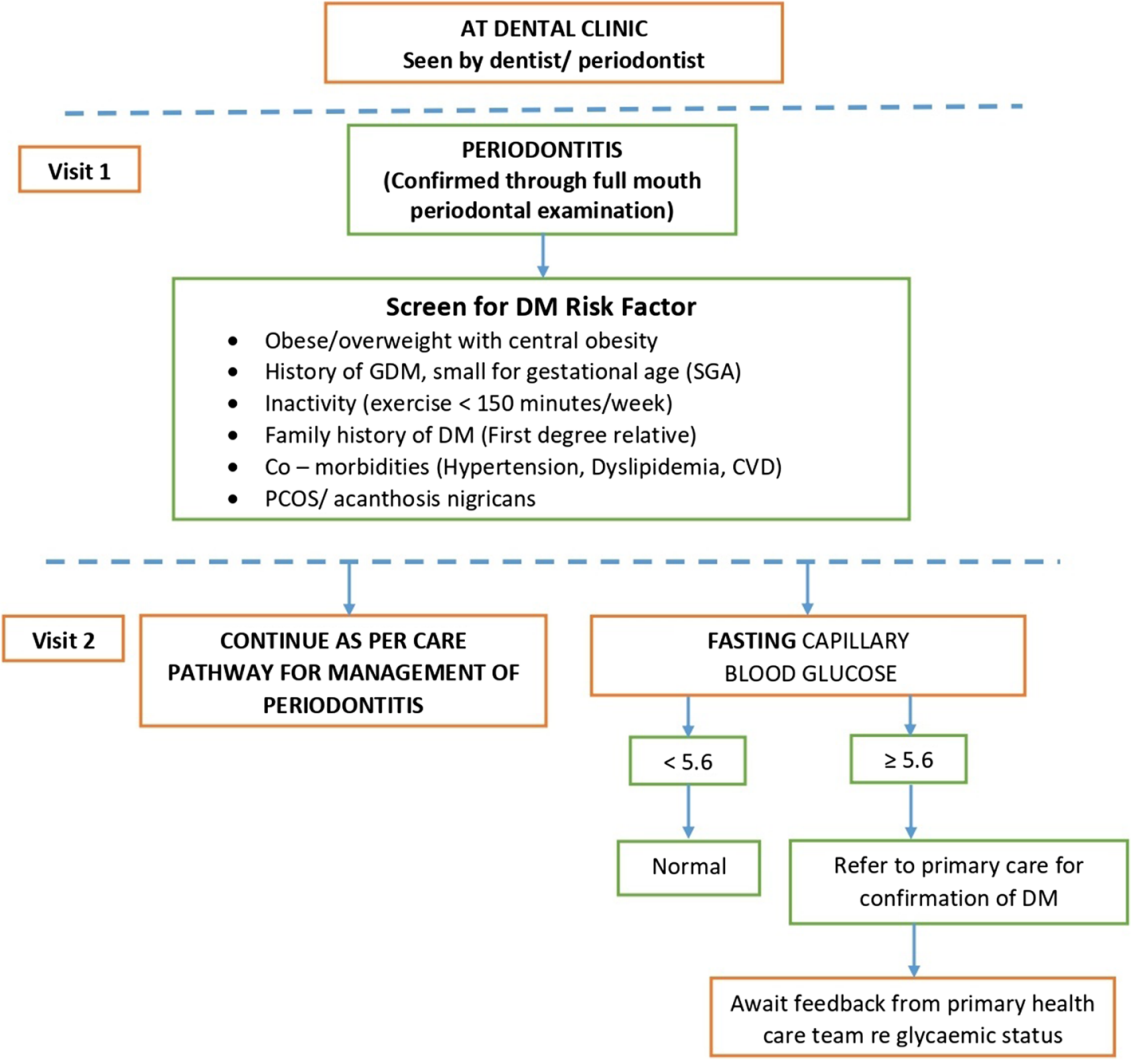
CODAPT-My^©^ Screening algorithm for the dental practitioner

This care pathway was designed to screen patients who presented with symptoms and signs of periodontitis to any dental care practitioner (Fig. 2). History taking should include screening for possible diabetes or prediabetes by identifying other risk factors such as obese or overweight with central obesity, history of gestational diabetes mellitus (GDM), inactivity (exercises < 150 min per week), family history of diabetes (among first degree relatives), hypertension, dyslipidaemia, polycystic ovarian syndrome (PCOS), acanthosis nigricans or small for gestational age.

Based on the current evidence and considering the national prevalence of diabetes and periodontitis, all patients diagnosed with periodontitis are recommended to undergo an evaluation of their glycaemic status. This screening procedure is best achieved by a fasting capillary plasma glucose level which can be scheduled from the second visit onwards or at least once while undergoing treatment at the dental clinic. If facilities are available for a venous plasma glucose testing, then this method should be employed.

The dental practitioner must obtain information or verify the glycaemic status of each patient suspected or confirmed to have periodontitis. The cut off point for referral to a primary healthcare practitioner is a fasting capillary plasma glucose level ≥ 5.6 mmol/L. The referral is to confirm and initiate the appropriate treatment for prediabetes or diabetes. This recommendation is made to ensure that individuals with periodontitis who are unaware of their prediabetes or diabetes status are provided with the opportunity to receive adequate and timely medical intervention to reduce diabetes-related morbidity and mortality.

### The CODAPT-My^*©*^ algorithm for the primary healthcare practitioner

**Fig. 3:**
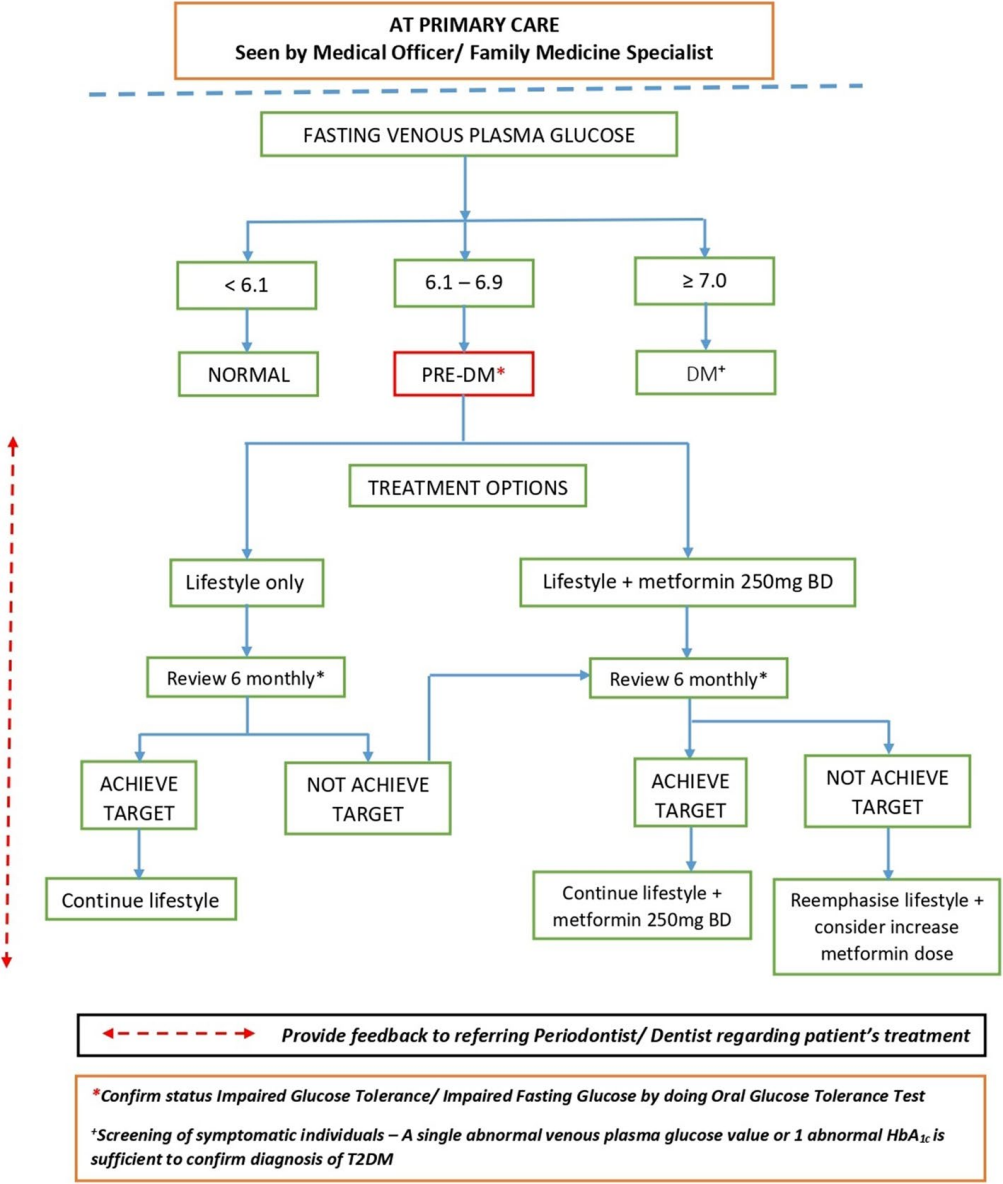
CODAPT-My^©^ Care algorithm for the primary healthcare link with the dental team

Upon receiving the referral from the Dentists or Periodontists, the primary healthcare practitioner should proceed to confirm the glycaemic status of a symptomatic (i.e., periodontitis) prediabetic or diabetic patient (Fig. 3). Apart from re-confirming the risk profile of the patient, confirmatory testing should be performed using fasting plasma glucose levels. This procedure would also provide an opportunity for risk profiling of the patients in terms of risk for cardiovascular disease (i.e., coronary heart disease or cerebrovascular events or subclinical heart disease).

At the time of writing, the Ministry of Health Malaysia had issued the sixth edition of the Clinical Practice Guidelines (CPG) Management of Type 2 diabetes mellitus in 2020 [20]. This issue emphasised the bidirectional relationship between periodontal disease and diabetes, calling for closer collaboration between physicians and oral healthcare professionals to improve glycaemic control. The CPG recommends that all physicians or medical health professionals should rule out periodontal disease in all newly diagnosed diabetic patients and if present, prompt referral to dentists is advised. However, the recommendation did not address measures to link patients with proven periodontal disease who are seen by Dentists/ Periodontists to confirm the glycaemic status of the patients. Hence, this warrants the need for CODAPT-My^©^ to address this gap.

**Fig. 4:**
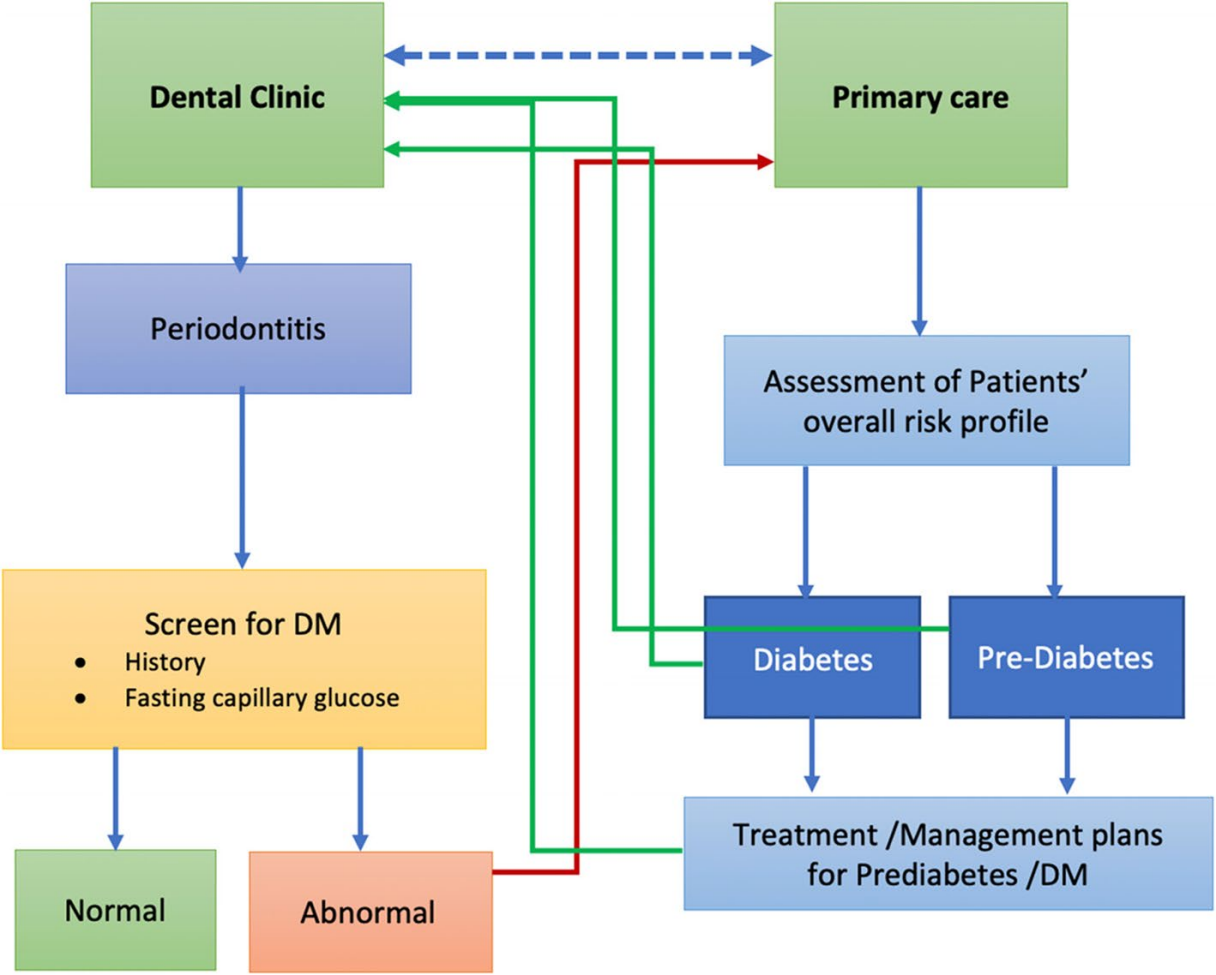
The CODAPT-My^©^: Shared care initiatives between dental and primary care services in Malaysia

This section of the CODAPT-My^©^ outlines the steps to be taken by the primary healthcare physician upon confirmation of glycaemic status, especially for those in the prediabetes group. Options for monitoring of the prediabetics are clearly mentioned to guide the monitoring plan. However, the most important aspect of this care pathway is the inclusion of the reminder prompt to update the referring Dentists/ Periodontists. A multidisciplinary care approach involving knowledge sharing, diabetic patients’ awareness to utilise dental services, and routine referral from the primary healthcare provider to the dental team should be advocated [16]. The overall overview of the CODAPT-My^©^ which illustrates the entire process from the periodontist/ dental clinic to the primary healthcare clinic is illustrated in Fig. 4.

## Discussion

To our knowledge, the CODAPT-My^©^ is the first care pathway which attempts to link bidirectional access between dental and primary healthcare services with the aim of providing coordinated and timely care for patients with deranged blood glucose control, specifically for patients who may be categorised as prediabetes or diabetes. The main aim of creating this pathway is to acknowledge periodontitis as a possible early sign of diabetes and to optimise the management of these patients within the current healthcare system, as recommended by the World Health Organization (WHO) [21]. The added benefit of the CODAPT-My^©^ is that it provides an opportunistic advantage for early identification of dental clinic attendees who are at risk of diabetes but are unaware of their current glycaemic status. Incidentally, the National Health and Morbidity Survey in 2019 found that 8.9% of diabetics were not aware of their diagnosis [1]. It is hoped that with the confirmation of the glycaemic status of patients presenting with periodontitis, more people will be able to make informed decisions on their health behaviour after knowing their diagnosis despite being asymptomatic, especially in the case of individuals who are prediabetes. This in turn will enable preventive measures to halt the progression of the disease.

The CODAPT-My^©^ pathway assigns specific roles and tasks for multidisciplinary shared care initiatives, namely among primary healthcare, dental as well as clinical pharmacy management. The acknowledgement of the increasing role of clinical pharmacists in managing patients with diabetes adds inclusivity and more efficient clinical management of these patients. Our pathway components were based on current available resources and took into consideration limitations in public healthcare facilities. Like most care pathways, this pathway aims to improve the current healthcare practices by optimising available resources, and it is desirable for an economic evaluation to follow suit to assess its cost effectiveness in comparison to conventional care practices. In cases of non-communicable diseases management such as diabetes and periodontitis, measures to contain costs should be made a priority, without jeopardising the clinical outcomes. Several studies have demonstrated that the costs of diabetes management were lower when patients also received periodontal therapy [22, 23].

In a UK study, the cost-effectiveness of non-surgical periodontal therapy and rigorous maintenance treatment in patients with type 2 diabetes and periodontitis was evaluated and it was found that periodontal therapy is cost-effective for diabetes patients with controlled HbA_1c_ [24]. Of relevance, there are Malaysian studies on care pathways which have estimated the potential benefits of periodontitis management and other NCD-related management (i.e., stroke); therefore, we anticipate that the addition of CODAPT-My^©^ and its acceptance by local clinicians would further optimise patients’ clinical outcomes and improve efficiency of the local public healthcare system [25–27].

One of the challenges in multidisciplinary or transdisciplinary management is the acceptance of shared care initiatives by clinicians of different disciplines. It has been well documented that clinician behaviours are unpredictable and difficult to change [25, 28]. Not all clinicians embrace inclusivity, and some are more comfortable to practice within ‘silos’, believing that transdisciplinary management invades disciplinary boundaries. In addition, issues of poor communication which commonly occur with interprofessional referrals and consultation have become a risk management concern [28]. These issues need to be resolved in order to enhance patient-centred approach to diabetes care within the existing healthcare system navigation.

Bisset and colleagues in the UK [19] reported that since there was negative interprofessional feedback about treating periodontitis patients with diabetes, a measure of compromise was recommended involving patient-driven prompting (signposting) which may serve to avoid the friction among clinicians. However, this patient-driven strategy would require a good level of health literacy related to understanding of diabetes care and self-monitoring among the patients. Considering that one third of the Malaysian population have poor health literacy and lack the impetus for self-monitoring of illnesses, this approach would not yet be appropriate for adoption in Malaysia [1, 26]. Nonetheless, in relation to dental service utilisation, the low dental uptake among Malaysian adults could be improved after receiving a prompt triggered by the medical practitioner. This consequently could increase the dental attendance of patients with NCD, such as those with type 2 diabetes.

By synergistically promoting oral health, controlling for risk factors, and referring for timely dental care, primary healthcare doctors can potentially play an integral role in strengthening the integration between NCD and oral health management [29]. Unfortunately, where systems are not in place to create this networking between disciplines, it would then be up to individual providers or professional organisations to develop these inter-professional relationships. However, these cross discipline relationships are often time consuming, inefficient, and unsustainable for the patient and the providers. Hence, the implementation and nation-wide acceptance of CODAPT-My^©^ provides a huge potential for establishing networking within the public healthcare facilities and eventually, referral systems across private healthcare facilities. The main advantage and added value of this pathway are that it was developed via a consensual process between medical, dental and pharmacy practitioners from the Ministry of Health and the private sectors including those in university settings. Future direction would be to evaluate the acceptance of CODAPT-My^©^ uptake nationwide and to evaluate its impact on clinical outcomes of periodontitis patients with diabetes and vice-versa.

## Conclusion

The CODAPT-My^©^ care pathway has the potential to link dental clinics with primary healthcare in diagnosing and/or optimising treatment of prediabetes/diabetes among patients receiving periodontitis treatment.

## Data Availability

The datasets used and analysed during the current study are available from the corresponding author on reasonable request.

## References

[1] Institute for Public Health (2020). National Health and Morbidity Survey (NHMS) 2019: non-communicable diseases, healthcare demand, and health literacy—key findings.

[2] American Diabetes Association (2020). 3. Prevention or delay of type 2 diabetes. Standards of medical care in diabetes—2021. Diabetes Care.

[3] Peres MA, Macpherson LMD, Weyant RJ, Daly B, Venturelli R, Mathur MR, Listl S, Celeste RK, Guarnizo-Herreño CC, Kearns C (2019). Oral diseases: a global public health challenge. The Lancet.

[4] Nazir M, Al-Ansari A, Al-Khalifa K, Alhareky M, Gaffar B, Almas K (2020). Global prevalence of periodontal disease and lack of its surveillance. Sci World J.

[5] Kassebaum NJ, Bernabé E, Dahiya M, Bhandari B, Murray CJL, Marcenes W (2014). Global burden of severe periodontitis in 1990–2010: a systematic review and meta-regression. J Dent Res.

[6] Oral Health Division, Ministry of Health Malaysia. National Oral Health Survey of Adults (2010). Putrajaya; 2014.

[7] Mohd-Dom T, Abd Muttalib K, Ayob R, Yaw SL, Mohd-Asadi AS, Abdul Manaf MR, Aljunid SM (2013). Periodontal status and provision of periodontal services in Malaysia: trends and way forward. Malaysian J Public Health Med.

[8] Tan YR, Tan EH, Jawahir S, MohdHanafiah AN, MohdYunos MH (2021). Demographic and socioeconomic inequalities in oral healthcare utilisation in Malaysia: evidence from a national survey. BMC Oral Health.

[9] Graziani F, Gennai S, Solini A, Petrini M (2018). A systematic review and meta-analysis of epidemiologic observational evidence on the effect of periodontitis on diabetes an update of the EFP-AAP review. J Clin Periodontol.

[10] Genco RJ, Graziani F, Hasturk H (2020). Effects of periodontal disease on glycemic control, complications, and incidence of diabetes mellitus. Periodontology 2020.

[11] Khader YS, Dauod AS, El-Qaderi SS, Alkafajei A, Batayha WQ (2006). Periodontal status of diabetics compared with nondiabetics: a meta-analysis. J Diabetes Complications.

[12] Engebretson S, Kocher T (2013). Evidence that periodontal treatment improves diabetes outcomes: a systematic review and meta-analysis. J Clin Periodontol.

[13] Madianos PN, Koromantzos PA (2018). An update of the evidence on the potential impact of periodontal therapy on diabetes outcomes. J Clin Periodontol.

[14] Chen YF, Zhan Q, Wu CZ, Yuan YH, Chen W, Yu FY, Li Y, Li LJ (2021). Baseline HbA1c level influences the effect of periodontal therapy on glycemic control in people with Type 2 diabetes and periodontitis: a systematic review on randomized controlled trails. Diabetes Therapy.

[15] Khaw KT, Wareham N, Bingham S, Luben R, Welch A, Day N (2004). Association of hemoglobin A1c with cardiovascular disease and mortality in adults: the European prospective investigation into cancer in Norfolk. Ann Intern Med.

[16] Sahril N, Aris T, Mohd Asari AS, Yaw SL, Saleh NC, et al. Oral health seeking behaviour among Malaysians with type II diabetes. J Public Health Aspects. 2014;1(1):8. 10.7243/2055-7205-1-1.

[17] Ministry of Health, Malaysia. Health Facts 2020. Ministry of Health Planning Division Health Informatics Centre. 2020;1–19.

[18] McGowan K, Phillips T, Gielis E, Dover T, Mitchell G, Mutch A, Sexton C, Sowa P, Ivanovski S (2021). Developing a prototype for integrated dental and diabetes care: understanding needs and priorities. Aust Dent J.

[19] Bissett SM, Preshaw PM, Presseau J, Rapley T (2020). A qualitative study exploring strategies to improve the inter-professional management of diabetes and periodontitis. Prim Care Diabetes.

[20] Malaysian Endocrine Society, Ministry of Health Malaysia, Academy of Medicine Malaysia, Family Medicine Specialist Association of Malaysia (2020). Clinical practice guidelines management of type 2 diabetes mellitus.

[21] Petersen PE, Ogawa H (2005). Strengthening the prevention of periodontal disease: the WHO approach. J Periodontol.

[22] Smits KPJ, Listl S, Plachokova AS, Van der Galien O, Kalmus O (2020). Effect of periodontal treatment on diabetes-related healthcare costs: a retrospective study. BMJ Open Diabetes Res Care.

[23] Nasseh K, Vujicic M, Glick M (2017). The relationship between periodontal interventions and healthcare costs and utilization. Evidence from an integrated dental, medical, and pharmacy commercial claims database. Health Econ.

[24] Solowiej-Wedderburn J, Ide M, Pennington M (2017). Cost-effectiveness of non-surgical periodontal therapy for patients with type 2 diabetes in the UK. J Clin Periodontol.

[25] Zhu Y, Close K, Zeldin LP, White BA, Rozier RG (2019). Implementation of oral health screening and referral guidelines in primary health care. JDR Clin Trans Res.

[26] Amal N, Paramesarvathy R, Tee G, Gurpreet K, Karuthan C (2011). Prevalence of chronic illness and health seeking behaviour in Malaysian population: results from the Third National Health Morbidity Survey (NHMS III) 2006. Med J Malaysia.

[27] Mohd-Dom T, Mohd-Said S, Ooi YH, Zainuddin S, Yaacob M, Ramli H, Noor E, Abdul Aziz A (2021). The impact of a clinical pathway on treatment outcomes of patients with periodontitis in Public University Settings: a quasi-experimental study. Teikyo Med J.

[28] Atchison K, Rozier R, Weintraub J. Integration of oral health and primary care: communication, coordination and referral. NAM Perspectives. Washington, DC: National Academy of Medicine; 2018. p. 8.

[29] Kapoor S, Mohanty V, Balappanavar AY, Chahar P, Rijhwani K (2022). Primary health care workforce in Southeast Asia Region, existing status and strategies for non-communicable diseases and oral health alliance: a scoping review. Cureus.

